# Comparing percutaneous coronary intervention and coronary artery bypass grafting for left main stenosis on the basis of current regional registry evidence

**DOI:** 10.1016/j.xjon.2024.09.025

**Published:** 2024-10-01

**Authors:** Hristo Kirov, Tulio Caldonazo, Aryan D. Khayyat, Panagiotis Tasoudis, Johannes Fischer, Angelique Runkel, Murat Mukharyamov, Torsten Doenst

**Affiliations:** a Department of Cardiothoracic Surgery, Friedrich-Schiller-University Jena, Jena, Germany; b Division of Cardiothoracic Surgery, University of North Carolina, Chapel Hill, NC

**Keywords:** percutaneous coronary intervention, coronary artery bypass grafting, left main coronary disease

## Abstract

**Objectives:**

There is an ongoing debate whether percutaneous coronary intervention (PCI) or coronary artery bypass grafting (CABG) is the better choice for treatment of left main (LM) stenosis. We aimed to provide external validation for the recently reviewed guideline recommendations for invasive LM therapy by evaluating the impact of CABG or PCI on long-term survival from local reports of different regions in the world. We performed a systematic review and meta-analysis to address contemporary registry studies comparing PCI and CABG for patients with LM stenosis.

**Methods:**

Three databases were assessed. Our primary end point was long-term all-cause mortality. Secondary end points were major adverse cardiovascular events (MACE), myocardial infarction, repeat revascularization, stroke, and periprocedural mortality. Reconstruction of time-to-event data was performed.

**Results:**

A total of 2477 studies were retrieved. Seven studies with risk-adjusted populations were selected for the analysis. Four studies favored CABG and 3 studies showed no difference for the primary end point. Compared with PCI, patients who underwent CABG had lower risk of death (hazard ratio, 1.15; 95% confidence interval, 1.05-1.26, *P* < .01) and MACE (hazard ratio, 1.54; 95% confidence interval, 1.40-1.69, *P* < .01) during follow-up. Moreover, PCI was associated with more myocardial infarction, repeat revascularization, but less strokes when compared with CABG. There was no significant difference regarding periprocedural mortality. The MACE rate was lower after CABG in both early and late phase, which outweighs the higher rate of periprocedural stroke after CABG.

**Conclusions:**

Regional registry evidence supports the current notion of superior long-term endpoints with CABG compared with PCI for the treatment of LM stenosis over time.

**Figure:**
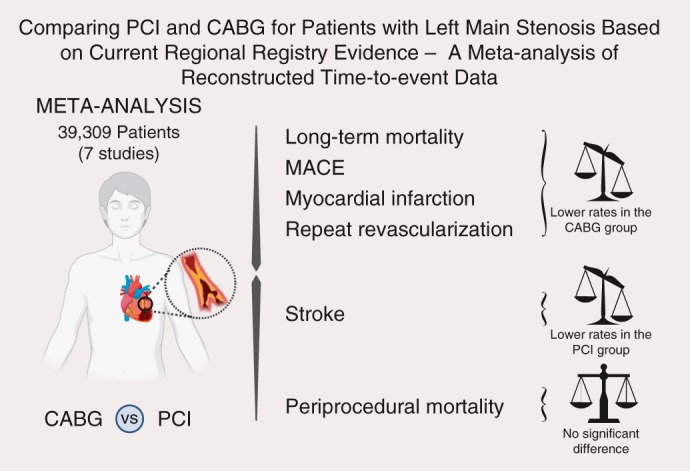
Summary of the main findings. *MACE*, Major adverse cardiovascular events.

**Central Message**

In terms of survival and MACE rate, regional registries confirm the current understanding that CABG generates better long-term results than PCI with comparable periprocedural mortality.

**Perspective**

This meta-analysis of regional registry data provides external validation of recently reviewed guideline recommendations of left main stenosis treatment and underscores the notion that CABG provides the best long-term results, where lower mortality was associated with less MI and MACE. This finding should receive more attention in heart team discussions when left main disease is present.

Left main coronary artery disease (LMCD), defined as visually determined stenosis of at least 50% in diameter, occurs in 4% to 7% of all patients undergoing coronary angiography and represents the greatest-risk lesion possible.^1^^,^^2^ Consequently, it is not surprising that patients with LMCD receiving isolated medical treatment exhibit a mortality rate of approximately 50% during the first 5 years after diagnosis.^3^ These numbers illustrate the importance of mechanical tools aimed at eliminating the thread from these coronary lesions.

In the past, coronary artery bypass grafting (CABG) emerged as the gold standard for the treatment of LMCD. However, with the rapid evolution of transcatheter interventions in recent decades, percutaneous coronary intervention (PCI) has become a frequently practiced alternative.^4^

In 2009, PCI appeared for the first time in the guideline recommendations of the American College of Cardiology, American Heart Association, and Society for Cardiovascular Angiography and Interventions as a Class IIb recommendation for LMCD and anatomically eligible lesions.^5^ Advances in stent technology, including functional imaging and the development of adjunctive pharmacotherapy, allowed more widespread application.^1^ Importantly, a number of randomized controlled trials (RCTs) comparing CABG and PCI for the treatment of LMCD were conducted^6^, ^7^, ^8^, ^9^ and have provided growing evidence for decision-making. The provided data not only led to several changes in guideline recommendations over time^10^ but also caused a prominent disagreement about these recommendations, which was recently resolved by the publication of a joint review of the left main (LM) section of the current revascularization guidelines of the European Society of Cardiology and European Association for Cardio-Thoracic Surgery.^10^

Although RCTs represent the highest quality of evidence and are regarded as the most rigorous way of determining the effect of an intervention on a clinical outcome,^11^^,^^12^ outcomes of RCTs generally reflect the average treatment effect often generated in samples from a very selected patient population.^13^ As a consequence, generalizations of trial findings outside the study population have been questioned.^14^ In contrast, registry studies, although likely burdened with various biases, may be considered to reflect the regional outcomes for a large fraction of the affected patient population in that region and therefore may provide information on the results of the available treatment modalities within the individual regions.^15^^,^^16^ This information is not provided by randomized trials, and the data from these registries may serve as external validation of outcomes of RCTs.^11^^,^^15^^,^^16^

On the basis of the aforementioned considerations, we aimed to provide external validation for the recent 2023 recommendations review for invasive LM therapy^17^ by evaluating meta-analytically the impact of CABG or PCI on long-term survival on the basis of local registry reports of different regions in the world. We applied the logic that if randomized evidence is selected and does not reflect “real life,” differences in regional care and outcomes should provide more heterogeneous outcomes compared with randomized trials.

## Methods

Ethical approval of this analysis was not required, as no human or animal subjects were involved. This review was registered with the National Institute for Health Research International Registry of Systematic Reviews (PROSPERO, CRD42023451201).

### Search Strategy

We performed a comprehensive literature search to identify contemporary studies reporting short- and long-term end points between PCI and CABG in patients with LMCD. Searches were run in August 2022 in the following databases: Ovid MEDLINE; Web of Science, and The Cochrane Library. The search strategy for Ovid MEDLINE is available in Table E1.

### Study Selection

The study selection followed the Preferred Reporting Items for Systematic Reviews and Meta-Analyses strategy. After de-duplication, records were screened by 2 independent reviewers (T.C. and A.K.). Any discrepancies and disagreements were resolved by a third author (H.K.). Titles and abstracts were reviewed against predefined inclusion and exclusion criteria.

### Eligibility Criteria

Studies were considered for inclusion if they were written in English and reported direct comparison of end points between populations undergoing PCI and CABG for LMCD. The inclusion also required the availability of reported registry results, with a specific focus on all-cause mortality or survival as the reported end point of interest. In addition, patients with nonisolated LMCD could be included in the reported series.

Exclusion criteria were non-English publications, studies lacking end points of interest, conference abstracts and proceedings, case reports, and noncomparative study designs. In addition, RCTs were excluded from consideration. Multinational cohorts or registries without clear data on geographic location were also excluded. The full text was pulled for a second round of eligibility screening. References of the selected articles were also reviewed for relevant studies not captured by the original search. The quality of the included studies was assessed using the Newcastle-Ottawa Scale (Table E2).

Two reviewers (T.C. and A.K.) independently performed data extraction. Accuracy was verified by a third author (H.K.). The extracted variables included study characteristics (publication year, country, sample size, study design, enrollment start and end dates, follow-up, presence or absence from population adjustment, and reported end points) as well as patient demographics (age, sex, mean left ventricular ejection fraction, hypertension, diabetes mellitus, smoking status, previous myocardial infarction [MI], previous PCI, body mass index, dialysis, chronic obstructive pulmonary disease, and peripheral vascular disease).

### End Points

Our primary end point was long-term survival. Secondary end points were major adverse cardiovascular events (MACE), MI, repeat revascularization, stroke, and periprocedural mortality. The individual study definitions of MACE and MI are described in Tables E3 and E4, respectively.

### Statistical Analysis

We conducted meta-analyses to compare the end points of PCI versus CABG in LMCD summarizing registry studies. We used reconstructed time-to-event data strategy for the primary end point and the occurrence of MACE.^18^^,^^19^ Odds ratios (ORs) and 95% confidence intervals (CIs) were calculated for the secondary end points of MI, repeat revascularization, stroke, and periprocedural mortality. The results are displayed in forest plots. An OR greater than 1 indicates that the end point is more frequently present in the PCI arm. Inherent clinical heterogeneity between the studies was balanced via the implementation of a random effects model. Between-study statistical heterogeneity was assessed with the Cochran Q statistic and by estimating *I*^2^. High heterogeneity was confirmed with a significance level of *P* < .10 and *I*^2^ of at least 50% or more. All statistical analyses were performed using R (version 4.3.1; R Project for Statistical Computing) within RStudio and STATA IC17.0 (StataCorp LLC).

### Individual Patient Survival Data Meta-Analysis

We used the methods described by Wei and colleagues to reconstruct individual patient data from the Kaplan-Meier curves of all eligible studies for the long-term end points.^18^^,^^19^ Raster and vector images of the Kaplan-Meier survival curves were preprocessed and digitized, so that the values reflecting to specific time points with their corresponding survival/mortality information could be extracted. Where additional information (eg, number-at-risk tables or total number of events) was available, it was used to further calibrate the accuracy of the time to events. Departures from monotonicity were detected using isotonic regression and corrected with a pool-adjacent-violators algorithm.^18^^,^^19^ To confirm the quality of the timing of failure events captured, we thoroughly checked the consistency with the reported survival or morality data provided in the original publications.

### Meta-Analysis of Reconstructed Data: One-Stage Survival Meta-Analysis

The Kaplan-Meier method was used to calculate the overall long-term survival as well as the frequency of MACE. The Cox proportional hazards regression model was used to assess between-group differences. For these Cox models, the proportional hazards assumption was verified by plotting scaled Schoenfeld residuals, log-log survival plots, and predicted versus observed survival functions. We plotted survival curves using the Kaplan-Meier product limit method and calculated the hazard ratios (HRs) and 95% CIs of each group. An HR greater than 1 indicated that the endpoint was more frequently present in the PCI arm.

## Results

### Study Characteristics

**Figure 1:**
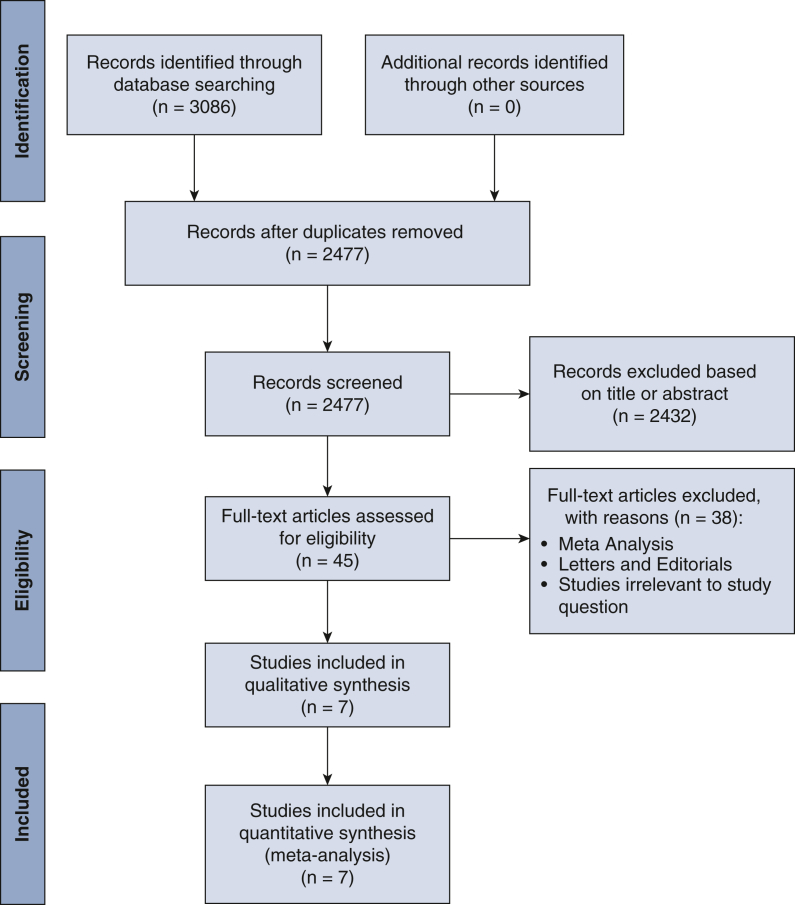
Preferred Reporting Items for Systematic Reviews and Meta-Analyses (*PRISMA*) flow diagram.

**Figure 2:**
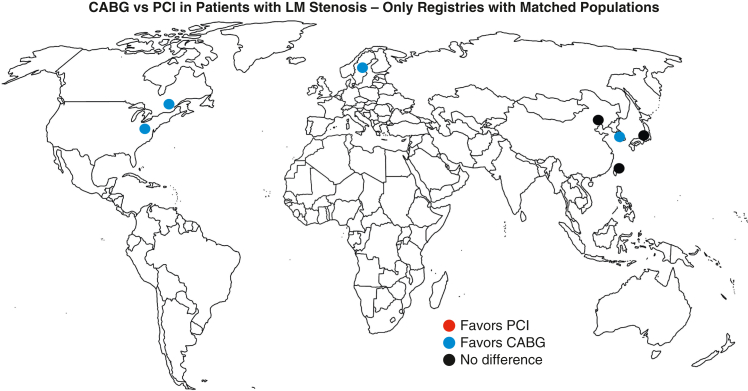
World map showing origin of the included studies. *CABG*, Coronary artery bypass grafting; *PCI*, percutaneous coronary intervention; *LM*, left main.

A total of 2477 studies were retrieved from the systematic search, of which 6 met the criteria for inclusion in the final analysis. Figure 1 shows the Preferred Reporting Items for Systematic Reviews and Meta-Analyses flowchart for study selection. Included studies were published between 2016 and 2023, all used registry data, and they originated from Sweden, Canada, the United States, Japan, Korea, China, and Taiwan. Figure 2 shows the geographic location of each registry study.

Table 1 shows the details of the included studies.^20^, ^21^, ^22^, ^23^, ^24^, ^25^, ^26^ All studies were determined on the basis of risk-adjusted populations. A total of 39,309 patients were included in the final analysis. The number of patients in each study ranged from 478 to 22,586.

**Table 1:** Summary of included studies

First author	Year of publication	Country	No. patients	Study design	Enrollment start and end dates	Follow-up, y	Population comparison
Persson et al^20^	2023	Sweden	11,137 1773 PCI, 9364 CABG	Retrospective, multicenter	January 2005 to December 2015	4.7	IPWA
Tam et al^21^	2023	Canada	22,586 1299 PCI, 21,287 CABG	Retrospective, multicenter	October 2008 to October 2020	7	PSM
Huckaby et al^22^	2022	USA	1091 193 PCI, 898 CABG	Retrospective, multicenter	January 2010 to January 2018	3.5	PSM
Yamamoto et al^23^	2021	Japan	855 383 PCI, 472 CABG	Retrospective, multicenter	January 2011 to December 2013	5.5	PSM
Park et al^24^	2018	Korea	2240 1102 PCI, 1138 CABG	Retrospective, multicenter	January 2000 to June 2006	12.0	PSM and IPWA
Yu et al^25^	2016	China	922 465 PCI, 457 CABG	Retrospective, single center	January 2003 to July 2009	7.1	PSM
Lu et al^26^	2016	Taiwan	478 208 PCI, 270 CABG	Retrospective, single center	January 2004 to December 2010	4.3	PSM

*PCI*, Percutaneous coronary intervention; *CABG*, coronary artery bypass grafting; *IPWA*, inverse probability weighting; *PSM*, propensity score matching.

### Patient Characteristics

Table E5 summarizes the demographic data of the patient population in each study. Age ranged from 62.0 to 73.6 years. Percentage of female patients ranged from 14.4% to 35.3%; mean left ventricular ejection fraction ranged from 49 to 64.3; percentage of hypertension ranged from 50.8% to 88.1%; percentage of diabetes mellitus ranged from 19.2% to 47.0%; percentage of current smokers ranged from 13.3% to 67.0%; percentage of previous MI ranged from 8.2% to 47.4%; percentage of previous PCI ranged from 5.2% to 35.1%; mean body mass index ranged from 23.5 kg/m^2^ to 28.7 kg/m^2^; percentage of previous dialysis ranged from 0.7% to 7.2%, percentage of chronic obstructive pulmonary disease ranged from 3.2% to 21.9%, and percentage of peripheral vascular disease ranged from 0.4% to 25.4%.

### Meta-Analysis

Table 2 provides a summary of end points from the included studies. Long-term survival was assessed in 6 studies^21^, ^22^, ^23^, ^24^, ^25^, ^26^ comprising 7100 patients, yielding an HR of 1.15 (95% CI, 1.05-1.26; *P* < .01). MACEs were evaluated in 4 studies^21^^,^^22^^,^^25^^,^^26^ with 4005 patients, resulting in an HR of 1.54 (95% CI, 1.40-1.69; *P* < .01). MI end points were reported in 6 studies involving 15,744 patients, with an OR of 1.88 (95% CI, 1.68-2.11; *P* < .01). Repeat revascularization was examined in 7 studies with 17,984 patients, showing an OR of 3.23 (95% CI, 2.47-4.22, *P* < .01). Stroke end points, analyzed in 6 studies with 15,744 patients, resulted in an OR of 0.64 (95% CI, 0.43-0.95, *P* = .03). Periprocedural mortality, determined by 4 studies comprising 14,220 patients, had an OR of 1.48 (95% CI, 0.94-2.33, *P* = .09).

**Table 2:** Outcomes summary

Outcome	Number of studies	Number of patients	Effect estimate (95% CI, *P* value)
Long-term survival	6	7100	HR, 1.15, 1.05-1.26, *P* < .01
MACE	4	4005	HR, 1.54, 1.40-1.69, *P* < .01
Myocardial infarction	6	15,744	OR, 1.88, 1.68-2.11, *P* < .01
Repeat revascularization	7	17,984	OR, 3.23, 2.47-4.22, *P* < .01
Stroke	6	15,744	OR, 0.64, 0.43-0.95, *P* = .03
Periprocedural mortality	4	14,220	OR, 1.48, 0.94-2.33, *P* = .09

*CI*, Confidence interval; *HR*, hazard ratio; *MACE*, major adverse cardiac events; *OR*, odds ratio.

### Individual Patient Data and Survival Curve Reconstruction

Overall, 6 Kaplan-Meier curves were processed, digitalized, and reconstructed.^21^, ^22^, ^23^, ^24^, ^25^, ^26^ Using the previously described methodology, we extracted the individual patient data from these curves. The entire observation period was 10 years. The average and the median pooled follow-up were 3.6 and 3.0 years, respectively.

**Figure 3:**
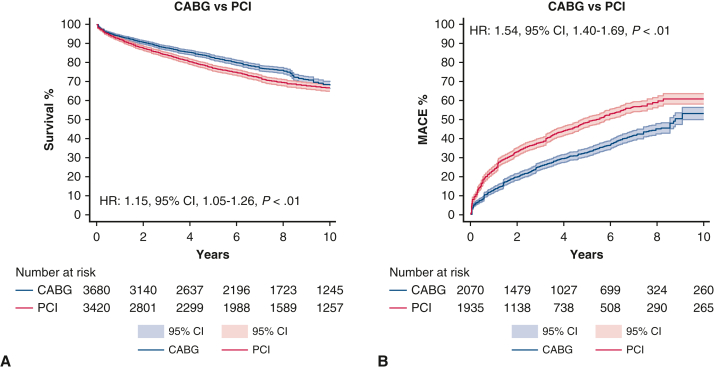
A, Pooled Kaplan-Meier curves showing long-term survival after PCI and CABG. B, Pooled Kaplan-Meier curves showing risk of MACE after PCI and CABG. *PCI*, Percutaneous coronary intervention; *CABG*, coronary artery bypass grafting; *MACE*, major adverse cardiac events.

Figure 3, *A*, shows the pooled Kaplan-Meier curves for the entire observation period for long-term survival. The patients who underwent CABG showed greater long-term survival when compared with the PCI group (HR, 1.15; 95% CI, 1.05-1.26, *P* < .01). Figure 3, *B*, shows the pooled Kaplan-Meier curves for the entire observation period for the occurrence of MACE. The patients who underwent CABG had significantly lower risk of MACE when compared with the PCI group (HR, 1.54; 95% CI, 1.40-1.69, *P* < .01).

**Figure E1:**
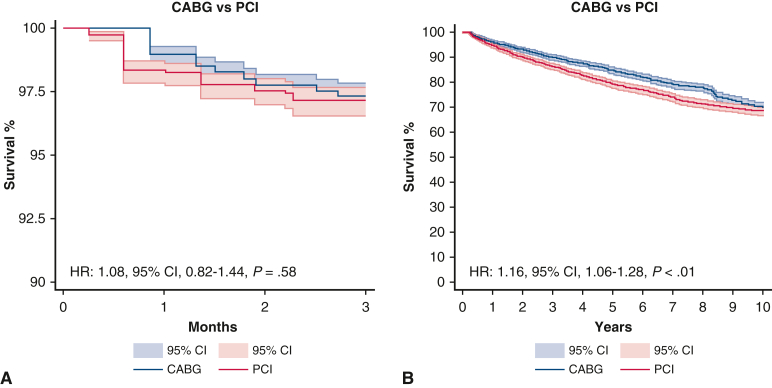
Landmark analysis showing the survival rates from 0 to 3 months (A) and from 3 months to 10 years (B). *CABG*, Coronary artery bypass grafting; *PCI*, percutaneous coronary intervention; *HR*, hazard ratio; *CI*, confidence interval.

**Figure E2:**
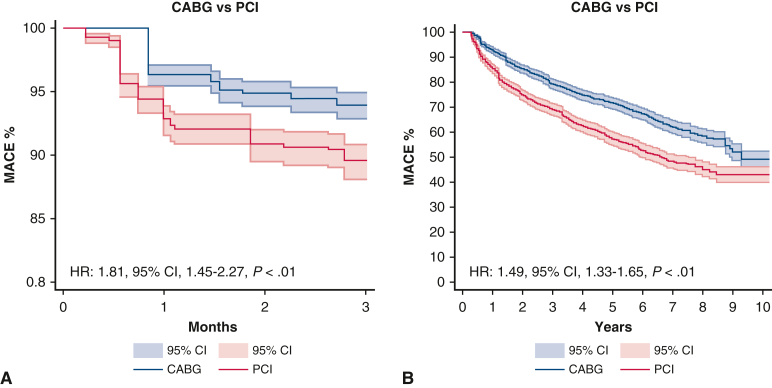
Landmark analysis showing the major adverse cardiovascular events rates from 0 to 3 months (A) and from 3 months to 10 years (B). *CABG*, Coronary artery bypass grafting; *PCI*, percutaneous coronary intervention; *HR*, hazard ratio; *CI*, confidence interval.

To investigate in depth the immediate periprocedural morbidity from the long-term effects of both procedures as therapeutic strategies, a landmark analysis using 3 months as split time point was performed. The analysis proved the robustness of the results, showing similar findings for the periprocedural endpoints in examination with absolute numbers/odds ratios, and also in time-to-event analysis with HTs (Figures E1 and E2).

### Secondary End Points

**Figure 4:**
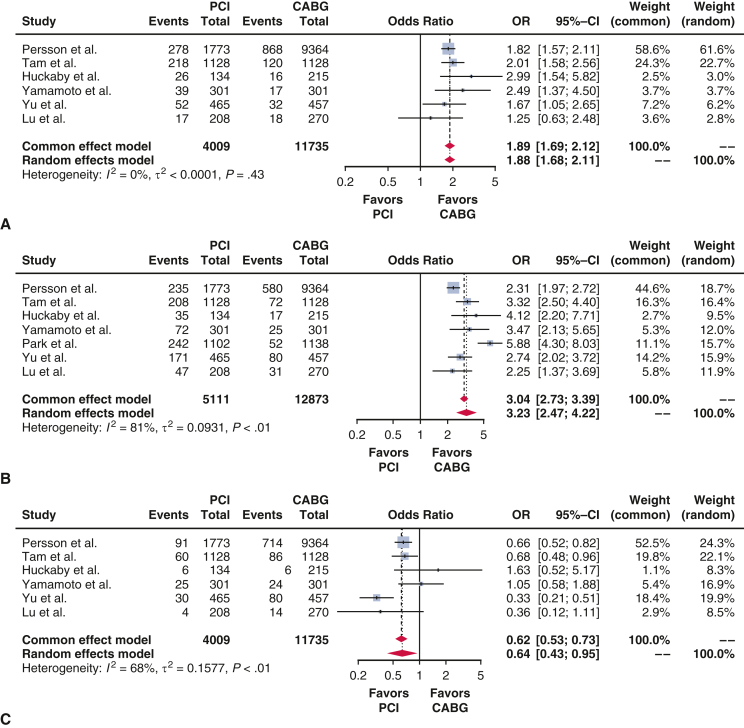
A, Forest plot for myocardial infarction. B, Forest plot for repeat revascularization. C, Forest plot for stroke. *PCI*, Percutaneous coronary intervention; *CABG*, coronary artery bypass grafting; *OR*, odds ratio; *CI*, confidence interval.

**Figure E3:**
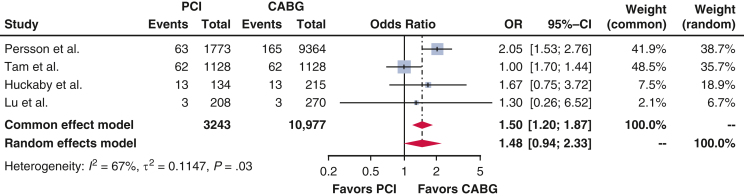
Forest plot for periprocedural all-cause mortality. *CABG*, Coronary artery bypass grafting; *PCI*, percutaneous coronary intervention; *HR*, hazard ratio; *CI*, confidence interval.

Figure 4, *A*, shows the forest plot for MI. The patients who underwent CABG showed lower incidence of MI when compared with the PCI group (OR, 1.88; 95% CI, 1.68-2.11, *P* < .01). Figure 4, *B*, shows the forest plot for repeat revascularization. The patients who underwent CABG showed lower incidence of re-revascularization when compared with the PCI group (OR, 3.23; 95% CI, 2.47-4.22; *P* < .01). Figure 4, *C*, shows the forest plot for stroke. The patients who underwent CABG showed greater incidence of stroke when compared with the PCI group (OR, 0.64; 95% CI, 0.43-0.95; *P* = .03). For instance, the proportion of off-pump surgery in the individual studies is detailed in Table E6. Figure E3 shows the forest plot for periprocedural mortality. There was no significant difference between the 2 therapy groups (OR, 1.48; 95% CI, 0.94-2.33; *P* = .09).

## Discussion

We demonstrate in this analysis that regional registry evidence supports the current notion of superior long-term outcomes with CABG compared with PCI in patients with LM coronary disease. Importantly, besides the survival difference observed, the MACE rate was also lower in the patients who underwent CABG in both the early and late phase. This outweighs the greater rate of periprocedural stroke in the CABG group. The significant occurrence of MI and repeat revascularization in the PCI group were the main adverse events within the MACE perimeter that led to this result and outweighed the periprocedural stroke rate.

The outcomes support a common pattern of comparisons for CABG and PCI in chronic coronary artery disease (CAD) by showing not only better long-term survival with CABG but also a lower incidence of MI and repeat revascularization. Thus, our results support and thereby confirm the recent LM review of the European Society of Cardiology/European Association for Cardio-Thoracic Surgery task force suggesting a Class I A recommendation for CABG and Class IIa recommendation for PCI in LMCD, irrespective of the SYNTAX score, but conclude that both treatment options are clinically reasonable on the basis of available expertise and local operator volumes.^17^

Several factors should be considered when interpreting the present findings. Isolated LMCD represents only a small subset of CAD, and LMCD is much more often a lesion associated with the presence of multivessel disease, with the need for addressing the other coronary regions being present in greater than 50% of cases.^27^ It has been shown previously that plaque burden and not stenosis per se is the main factor associated with cardiovascular events (MI, for example) and death.^28^ Thus, it is possible that the difference in events between CABG and PCI in our analysis is mainly driven by events occurring at the peripheral lesions and not at the main stem itself. Guideline-conform PCI is focused on treating flow-limiting lesions only and the majority of MIs occur at nonflow-limiting stenosis.^16^^,^^29^ In contrast, additional to bypassing the LMCD lesion, CABG may prevent the occurrence of new MIs by bypassing most other coronary lesions and providing downstream “collateralization” to the grafted vessel.^29^ This collateral has the potential to prevent future MIs, which are mainly caused by rupture and thrombosis of plaques that are most often not flow-limiting or less frequently caused by vessel occlusions from progression of flow-limiting lesions.^29^^,^^30^ This mechanistic approach provides a plausible explanation of our present results. The concept is confirmed by the observation that the survival advantage associated with CABG is also associated with a reduction of MI and repeat revascularization, a pattern that has repeatedly been observed by other investigators.^29^, ^30^, ^31^, ^32^

On the basis of the aforementioned logic, PCI should be comparable with CABG in patients with lower anatomic complexity of CAD (ie, less peripheral CAD often reflected by relatively low SYNTAX scores), which is the basis for current guideline recommendations.^10^ This reasoning is based on the plausible assumption that PCI is able to prolong life in patients with LM disease (because CABG does and outcomes may be the same), although the direct comparison of PCI and conservative therapy has never been performed. Although the concept is convincing, it may be worth assessing outcomes on the basis of the complexity of the lesion, because PCI for complex bifurcation lesions is more difficult to perform than for simple shaft lesions. The equality of PCI and CABG has found meta-analytical support for shaft lesions but not for distal lesions,^33^ which has now also been recognized by the LM task force.^17^

Irrespective of the involved mechanisms, there is another important finding from our analysis that should be addressed. Similar to previous analyses of pooled randomized^34^ and nonrandomized^16^ data, the intuitive assumption that more invasive treatments carry greater risks of periprocedural mortality has not found confirmation in the present meta-analysis. As risk and benefit assessment is a cornerstone guiding surgical/interventional decision-making,^35^ this fact seems to be important for future heart-team discussions.

Finally, Figure 2 highlights that CABG demonstrated advantages in studies conducted in Western Europe and North America, whereas in the regions of Southeast Asia, neither method surpassed the other. In this context, it is noteworthy that despite similar diameters of the proximal segments of coronary arteries and the LM coronary artery, as well as atherosclerotic burden in matched cohorts,^36^ CABG was associated with greater mortality rates among population of Asian descent. This phenomenon could theoretically explain the convergence of outcomes of different types of invasive treatments in Southeast Asian countries. However, it is important not to overlook that the cumulative weight of Asian publications in our meta-analysis (11.4% of the overall study population) significantly lagged behind those from Europe and North America.

The novel value of this work is therefore the external validation of previous randomized trials through a meta-analytical approach using registry data from different countries and continents under real-life conditions. Such validation serves as an important and potentially valuable piece in the mosaic of scientific methods applied to the specific phenomenon, in our case, the treatment of LMCD. The results we provide important support for current treatment recommendations of the guidelines and demonstrate a repetitive pattern that shows a larger difference in treatment effect in real-life data than in the randomized evidence. In their meta-analysis, Sabatine and colleagues^37^ were unable to find a significant difference between CABG and PCI for LM disease at 5 years, but the trend (crossing of curves and separation in favor of CABG after 2 years) was visible. Thus, the repetitive demonstration of life-prolonging effects underscores treatment recommendation as first line for CABG, the more invasive of the 2 treatment methods. Given the fact that daily practice deviates from the guidelines on a larger scale, even repeating previous recognitions with new tools and data sets is novel and important.

Finally, although stroke rates were greater in the CABG group, there is growing evidence showing that the incidence of perioperative stroke has gone down over the last few years, especially in a stable elective setting, particularly in cases without aortic manipulation.^38^ For instance, there was no difference in stroke rate between CABG and PCI in FAME 3 (Fractional Flow Reserve vs Angiography for Multivessel Evaluation), in which the most important contemporary techniques were used in both arms of the study.^39^

In summary, it seems that both PCI and CABG are good treatment options for selected patients and decisions should be driven by the heart team and determined by clinical considerations. This argument is further supported by the fact that despite better survival and less MIs and repeat revascularization, there were greater periprocedural rates of stroke associated with CABG in our analysis, a finding that knowingly has to do with the use of cardiopulmonary bypass and crossclamping. Thus, individual decision-making, especially in patients at a greater risk of stroke seems to be necessary, possibly guiding such patients toward PCI or to expert centers with experience in no-aortic touch CABG procedures.^40^

### Study Strengths and Limitations

This is the first meta-analysis of reconstructed time-to-event data to address this important topic. However, this work has the intrinsic limitations of observational series, including the risk of methodological heterogeneity of the included studies and residual confounders. First, the significant heterogeneity of the MI definitions could influence the results especially in the periprocedural phase, as already described in other observational and randomized studies. Second, although most of the studies included coronary anatomy severity as a covariate in their statistical model, the individual studies did not provide the SYNTAX score from the respective series. In this context, a detailed quantification of the extension and complexity of CAD could not be addressed in depth.

## Conclusions

This work suggest that, on the basis of regional registry evidence, CABG appears superior to PCI for the treatment of LM stenosis over time, without being associated with greater periprocedural mortality. Importantly, besides the survival difference observed, the MACE rate was lower in the patients who underwent CABG, in both the early and late phase. This outweighs the greater rate of periprocedural stroke in the CABG group.

## Conflict of Interest Statement

The authors reported no conflicts of interest.

The *Journal* policy requires editors and reviewers to disclose conflicts of interest and to decline handling or reviewing manuscripts for which they may have a conflict of interest. The editors and reviewers of this article have no conflicts of interest.

## Figures and Tables

**Table E1:** Search strategy for Ovid MEDLINE

"left"[All Fields] AND "main"[All Fields] AND ("percutaneous coronary intervention"[MeSH Terms] OR ("percutaneous"[All Fields] AND "coronary"[All Fields] AND "intervention"[All Fields]) OR "percutaneous coronary intervention"[All Fields]) AND ("coronary artery bypass"[MeSH Terms] OR ("coronary"[All Fields] AND "artery"[All Fields] AND "bypass"[All Fields]) OR "coronary artery bypass"[All Fields] OR ("coronary"[All Fields] AND "artery"[All Fields] AND "bypass"[All Fields] AND "grafting"[All Fields]) OR "coronary artery bypass grafting"[All Fields])
Translations
**percutaneous coronary intervention:** "percutaneous coronary intervention"[MeSH Terms] OR ("percutaneous"[All Fields] AND "coronary"[All Fields] AND "intervention"[All Fields]) OR "percutaneous coronary intervention"[All Fields] **coronary artery bypass grafting:** "coronary artery bypass"[MeSH Terms] OR ("coronary"[All Fields] AND "artery"[All Fields] AND "bypass"[All Fields]) OR "coronary artery bypass"[All Fields] OR ("coronary"[All Fields] AND "artery"[All Fields] AND "bypass"[All Fields] AND "grafting"[All Fields]) OR "coronary artery bypass grafting"[All Fields]

**Table E2:** Assessment of risk of bias using the Newcastle Ottawa Scale

Study	Selection	Comparability	Outcome/exposure
Persson	****	**	***
Tam	****	**	***
Huckaby	**	**	*
Yamamoto	****	**	**
Park	****	**	***
Yu	****	**	***
Lu	****	**	***

**Table E3:** Major adverse cardiovascular events definition of the individual studies

Study	Definition
Tam	All-cause mortality, new hospital admission for MI, stroke, or repeat revascularization
Huckaby	All-cause mortality, stroke, MI, and repeat revascularization
Yu	Cardiac death, MI, stroke, or repeat revascularization
Lu	All-cause mortality, MI, stroke, and clinically driven repeat revascularization

*MI*, Myocardial infarction.

**Table E4:** Myocardial infarction definition of the individual studies

Study	Definition
Persson	Clinical definition for MI that relied on administrative codes obtained from discharge reports.
Tam	Clinical definition for MI that relied on administrative codes obtained from discharge reports.
Huckaby	Not described.
Yamamoto	To keep consistency with CREDO-Kyoto PCI/CABG registry Cohort-1 and -2, myocardial infarction was also adjudicated according to the Arterial Revascularization Therapies Study (ARTS) definition, in which only Q-wave myocardial infarction was regarded as myocardial infarction when it occurred within 7 days of the index procedure.
Park	Defined as the documentation of a new pathologic Q-wave after the index revascularization.
Yu	Periprocedural MI (<7 days after intervention) was defined as elevated serum creatinine kinase-MB isoenzyme 5 times the upper limit of normal after CABG and 3 times the upper limit of normal after PCI. MI after the periprocedural period was defined as any typical increase and decrease of biochemical markers of myocardial necrosis with 1 of the following: cardiac symptoms, development of Q waves on electrocardiography, or electrocardiographic changes indicative of ischemia.
Lu	Presence of significant new Q waves in at least 2 electrocardiographic leads or the presence of symptoms compatible with MI associated with an increase in creatine kinase-MB fraction 3 or more times the upper limit of the reference range.

*MI*, Myocardial infarction; *CABG*, coronary artery bypass grafting; *PCI*, percutaneous coronary intervention.

**Table E5:** Demographics of included patients from the selected studies

Study	Age, y, mean ± SD		Female, %		LVEF, mean ± SD		HP, %		DM, %		Current smoker, %		Previous MI, %		Previous PCI, %	
	PCI	CABG	PCI	CABG	PCI	CABG	PCI	CABG	PCI	CABG	PCI	CABG	PCI	CABG	PCI	CABG
Persson	72.8 ± 10.7	69.6 ± 8.8	28.2	21.0	NR	NR	64.3	63.8	19.2	22.0	17.2	15.8	19.1	16.3	7.5	5.2
Tam	73.6 ± 10.8	73.6 ± 10.6	32.7	34.2	NR	NR	87.7	87.9	45.7	44.1	13.3	15.6	31.4	33.2	NR	NR
Huckaby	74.4 ± 9.7	73.7 ± 8.2	35.1	35.3	49 ± 13.5	49.7 ± 14.9	88.1	87.9	41.04	41.9	16.7	17.6	46.3	47.4	35.1	34.9
Yamamoto	72.3 ± 9.7	70.2 ± 9.1	24.8	20.3	59.4 ± 14.1	61.6 ± 13.0	82.5	81.4	42.6	48.7	17.8	14.0	19.8	18.6	NR	NR
Park	62.6 ± 11.2	63.2 ± 9.7	28.4	30.6	59.7 ± 11.1	59.4 ± 11.5	50.8	50.8	29.9	31.3	28.5	27.2	10.2	8.2	15.0	14.7
Yu	62.0 ± 11.9	63.7 ± 9.7	21.2	17.5	64.3 ± 8.2	61.3 ± 10.4	61.5	58.9	30.8	28.7	NR	NR	14.2	21.7	14.6	6.8
Lu	70.0 ± 12.0	69.0 ± 11.0	15.9	14.4	49.0 ± 12.0	49.0 ± 12.0	78.0	83.0	47.0	46.0	50.0	67.0	NR	NR	NR	NR

Study	BMI, mean ± SD		Dialysis, %		COPD, %		PVD, %	
	PCI	CABG	PCI	CABG	PCI	CABG	PCI	CABG
Persson	26.4 ± 3.6	26.8 ± 3.6	1.0	0.7	5.1	3.2	0.4	0.7
Tam	NR	NR	7.0	5.0	20.6	21.9	12.0	11.7
Huckaby	27.7 ± 5.3	28.7 ± 5.4	5.2	5.1	NR	NR	25.4	24.2
Yamamoto	23.5 ± 3.5	23.5 ± 3.4	6.0	7.2	NR	NR	17.8	9.3
Park	NR	NR	2.4	3.2	NR	NR	2.3	1.5
Yu	NR	NR	NR	NR	NR	NR	5.2	7.4
Lu	NR	NR	NR	NR	NR	NR	13.0	16.0

*SD*, Standard deviation; *LVEF*, left ventricular ejection fraction; *HP*, hypertension; *DM*, diabetes; *MI*, myocardial infarction; *PCI*, percutaneous coronary intervention; *CABG*, coronary artery bypass grafting; *NR*, not reported; *BMI*, body mass index; *COPD*, chronic obstructive pulmonary disease; *PVD*, peripheral vascular disease.

**Table E6:** Proportion of off-pump surgery described in each study

Study	Off-pump CABG (%)
Persson	Not reported
Tam	Not reported
Huckaby	“Almost a quarter”
Yamamoto	58.3
Park	42.0
Yu	92.3
Lu	4.4

*CABG*, Coronary artery bypass grafting.
